# Improving detection and notification of tuberculosis cases in students in Shaanxi province, China: an intervention study

**DOI:** 10.1186/1471-2458-11-147

**Published:** 2011-03-04

**Authors:** Tianhua Zhang, Liujia Guo, Shaoru Zhang, Weiping Liu, Guanghua Chen, Ma Hui, Guangxue He, Marieke J van der Werf, Susan van den Hof

**Affiliations:** 1 Shaanxi Provincial Institute for TB Control and Prevention, Xi'an, Shaanxi province, PR China; 2 Medical school, Xi'an Jiao Tong University, Xi'an, Shaanxi province, PR China; 3 National Center for Tuberculosis Control and Prevention, Chinese Center for Disease Control and Prevention (CDC), Beijing, PR China; 4 KNCV Tuberculosis Foundation, The Hague, The Netherlands; 5 Center for Infection and Immunity Amsterdam (CINIMA), Academic Medical Center, University of Amsterdam, The Netherlands

## Abstract

**Background:**

Cooperation between different public and private health institutes involved in tuberculosis (TB) control has proven to enhance TB control in different settings. In China, such a mechanism has not been set up yet between Centers for Disease Control (CDCs) and university hospitals despite an increased TB incidence among students. This study aims to improve arrival of TB suspects identified by universities at the CDCs in order to manage them under standardized, directly observed treatment-short course (DOTS) conditions according to the National Tuberculosis Programme (NTP) guidelines.

**Methods:**

Five matched pairs of universities were randomly assigned to the control and intervention group. After a baseline survey, a cooperation mechanism between local CDCs and university hospitals was set up in the intervention group. The effects on referral of TB suspects to the local CDC, tracing by the local CDC, and arrival at the local CDCs were assessed. Differences were tested by means of the chi-square test.

**Results:**

During the baseline survey, the referral, tracing and arrival rates were between 37% and 46%. After implementation of the cooperation mechanism, these rates had not changed in the control group but increased significantly in the intervention group: the referral, tracing and arrival rates were 97%, 95%, and 93%, respectively.

**Conclusions:**

It is feasible and effective to set up cooperation between CDCs and university hospitals to increase the number of TB suspects examined by CDCs and increase the number of TB patients treated under DOTS conditions. These public-public mix (PPM) activities should be expanded to cover all other university hospitals in China.

## Background

Shaanxi province is located in the western region of China, and has a population of 36.7 million. In 2005, 39,822 TB cases were reported in Shaanxi, a notification rate of 109/100,000 population. Among 875,800 students in 72 universities in Shaanxi [1], the reported incidence was 143/100,000. In the last 10 years, several TB outbreaks in Chinese universities have been observed [2-6]. Therefore, TB in this special population is of concern and efforts should be made to improve TB control in universities.

In China, the National Tuberculosis Referring and Tracing System for the management of TB suspects and patients has been introduced in 2005. The National Tuberculosis Programme (NTP) guidelines stipulate that all medical institutions report TB suspects and patients in the internet based reporting system, and that they refer the suspects and patients to the TB dispensary at the local CDC for diagnosis and treatment [7]. The CDCs use smear examination and chest X-ray to diagnose TB or exclude it according to the standard of pulmonary TB diagnosis of Ministry of Health (WS288-2008) which is coherent with International Standards for TB Care [8]. TB patients and TB suspects who are registered in the internet based reporting system and do not reach the CDC clinics within three days, should be traced by CDC staff either by telephone or through home visits [7].

Recently, many efforts have been made in China to improve the cooperation between general hospitals and CDCs with the aim of strengthening TB patient referral and increasing the case detection [9]. No cooperative activities have been established yet between universities and TB dispensaries in China. According to the information in the internet based reporting system, 45% of patients reported as TB suspects by university hospitals in Shaanxi in 2005 were referred to the CDC (unpublished data). The arrival rate for those referred was 40%, so only 18% of TB suspects among university students arrived at the CDC. This is much lower than the reported average of 50% among all referred TB suspects in Shaanxi province.

The purpose of this intervention study was to improve referral and tracing of TB suspects identified by the university hospitals through cooperation between the university and CDCs in Shaanxi province. This will lead to an increased proportion of TB patients being treated correctly, under DOTS conditions.

## Methods

### Study design and study population

In China, almost every university has its own clinic, which provides medical care for common diseases, including small surgery and pharmacy services. Before initiating the intervention a survey was conducted to describe the baseline situation regarding TB control in the university hospitals. After the implementation of a cooperation mechanism between university hospital clinics and the local CDCs, the effects on the referral of TB suspects from university hospitals to the CDCs and tracing of TB suspects by the CDCs were assessed.

Inclusion criteria for universities were: having an in-patient clinic at the campus, having ≥ 5000 students, and willingness to participate as assessed before the start of the study. All but two of the 72 universities in Shaanxi province that fulfilled the inclusion criteria agreed to participate in this study: 47 universities in the provincial capital Xi'an, and 23 in other districts.

All students of the selected 10 universities in Shaanxi province that were registered at the universities between Jan.1, 2007 and Jan. 31, 2008 were eligible for inclusion in our study. Pulmonary tuberculosis suspects were defined as persons with cough, sputum production or haemoptysis for 2-3 weeks.

The five rates related to notification of university student TB suspects were referral rate, referral arrival rate, tracing rate, tracing arrival rate and total arrival rate. Table 1 shows the definitions and calculation methods of these rates.

**Table 1:** Formulae to calculate rates related to referral of TB suspects by the university clinics, tracing by, and arrival at the local Centers for Disease Control (CDCs)

Referral rate	Number of TB suspects referred by university hospital (= based on number of referral sheets)/Number of TB suspects registered through the internet based report system
Referral arrival rate	Number of TB suspects referred by university hospitals that arrived at the CDC within three days, without tracing/Number of TB suspects referred by university hospitals
Tracing rate	Number of TB suspects identified at the university hospitals reported through the internet based system, who did not reach the CDC in three days and were traced by the CDC according to the NTP requirement/Number of TB suspects identified at the university hospitals who were reported by the internet based system and did not arrive at the CDC in three days
Tracing arrival rate	Number of TB suspects at the university hospitals who arrived at the CDC after tracing/Number of TB suspects at the university hospitals who were traced by the CDC
Total arrival rate	Number of TB suspects at the university hospitals who arrived directly at the CDC plus those who arrived after tracing by the CDC/Number of TB suspects at university hospitals registered through the internet based report system

### Sampling

We wanted to be able to minimally detect an increase from 50% (P1) to 80% (P2) in any rate after initiation of the intervention. Using a level of significance of 95% (α = 1.96) and a power of 90% (β = 1.28) the required number of TB suspects was calculated using the formula: N = (1.96+1.28)^2 ^* (((P1*(1-P1))+(P2*(1-P2)))/(P1-P2)^2^. The required sample size was 48 suspects for the control and intervention group. An additional 20% was added to account for missing data, for a total of 58 suspects. We estimated that in half a year in five universities clinics, at least 58 TB suspects would present themselves.

First, three out of 47 universities were randomly sampled from Xi'an, and two out of 23 from other districts in the province. Thereafter, five universities were selected to match the sampled universities according to the location, type and size of the university. Of the matched pairs one was randomly allocated to the intervention group and the other to the control group.

### Study implementation

The study period was divided into two periods, the baseline period (January-July 2007), and the intervention period (August 2007-January 2008). During the baseline period, university hospital directors and doctors, and CDC directors and supervisors of the TB control departments within the CDC were interviewed on the current status of TB control activities within and between these institutes and were asked for recommendations on how to improve TB control in universities by means of a structured interview. Also, baseline referral, tracing, and arrival rates of TB suspects among university students were assessed. In China, pulmonary tuberculosis suspects are defined as persons with cough, sputum production or haemoptysis for 2-3 weeks. Based on findings from the baseline survey, an intervention plan was developed and implemented in August-September 2007 in the intervention group: a TB control program was set up in the university hospitals, and staff members were assigned specifically to TB control. The provincial CDC provided training to the hospital staff members on TB control (diagnosis, reporting, referral, management of TB cases, and health education to students). Hospital doctors received a small incentive of 20 RMB (≈2.5 USD) when they referred a TB suspect to the CDC, and this suspect was diagnosed with TB at the CDC. At the end of the baseline and intervention period, referral and arrival rates were calculated using data from the paper and electronic medical records at the university hospital and the CDC.

### Data-entry and data-analysis

Data were entered twice, by different persons, in EpiData v3.1 (Epidata Association, Denmark) and checked for discrepancies. The statistical software package SPSS version 13.0 (SPSS Inc, Chicago, USA) was used for data analysis. Differences between referral, tracing and arrival rates before and after the intervention were tested by means of the χ2-test.

### Quality Control

The selected universities did not know which other universities were included in the study. During the study period, no meetings were held for the staff of participating universities. Also, the participating universities were not very closely located to each other. Therefore, we consider it unlikely that the control universities were aware of the intervention that was implemented and as such we do not expect spillover effects from the intervention universities to other universities.

### Ethical considerations

Written informed consent was obtained from all TB suspects whom presented at the included university hospitals. The study was approved by the national ethical committee of operational research at the National Center for TB control. This study complied with the requirements of the Declaration of Helsinki.

## Results

### Characteristics of university hospitals and CDCs

All universities had a hospital at the main campus while five also had outpatient clinic(s) at one or more of their other campuses which were managed by the hospital. None of the hospitals could perform sputum smear examination but the hospitals in four universities had X-ray available for screening (Table 2). Five universities had campuses in different districts. Overall, the campuses were distributed among seven districts, 3 of which were in Xi'An city. Each district has its own CDC. Of the seven CDCs, five had a separate room for diagnosis of TB, a sputum smear test room, and X-ray room; and two cooperated with a hospital for TB diagnosis and treatment. In total there were 43 staff members for TB control in these seven CDCs (3-12 staff members per CDC).

**Table 2:** Characteristics of the study universities in the control and intervention group

University*	City	Category	Number of campuses	Number of CDCs	Health services		
						
					Hospital	Outpatient clinic	Number of students
A1	Xi'an	public	3	2/7	1	2	34000
A2	Xi'an	public	2	2/7	1	1	15000
A3	Xi'an	private	2	1/7	2	0	30000
A4	Yan'an	public	3	2/7	1	2	14000
A5	Han zhong	public	2	1/7	2	0	22000
B1	Xi'an	public	4	3/7	1	3	28000
B2	Xi'an	public	2	2/7	1	1	24000
B3	Xi'an	private	1	1/7	1	0	35000
B4	Yu lin	public	1	1/7	1	0	10000
B5	Shang luo	public	1	1/7	1	0	6000
Total			21	7	12	9	218000

* A1-A5: 5 universities in intervention group B1-B5: 5 universities in control group

1/7-3/7: numerator presents the number of CDC manage different campus of university

Denominator presents the total number of CDC

### Findings of interviews during the baseline period

Through interviews with university hospital and local CDC staff we identified some factors that impeded effective TB control for university students being implemented. CDC directors indicated that university hospital doctors were not trained on TB control due to limited human resources at the CDCs. Most hospitals had not assigned staff specifically for TB control tasks. No guidelines for patient registration, reporting, and referral were in place. University hospital staff was not familiar with NTP guidelines. Because of fear of spread and student panic in the campuses, the university usually asked university TB patients to suspend schooling and go home or to a hospital for treatment. These patients were never reported to the local CDC.

### Changes at the university hospital as a result of the intervention

During the baseline period, not all conditions for good quality TB control were in place (Table 3). In the intervention group, the situation had improved after the intervention period while in the control group the situation was still similar. Six out of seven university hospitals (4/5 universities) in the intervention group had set up a department of TB control, and all the hospitals had assigned a physician for TB control; had formulated guidelines on reporting and referral of TB suspects; had established a cooperation with the CDC; and had arranged health education activities on TB for students.

**Table 3:** TB control at the university hospitals before and after the intervention

	Before		After	
	
	Control	Intervention	Control	Intervention
	N = 5	N = 7	N = 5	N = 7
Number of hospitals with TB control program in place	2	2	2	6
Staff specifically assigned for TB control	1	1	1	7
System for reporting and referring TB suspects to CDC in place	0	0	0	7
Register for referral of TB suspects in place	0	0	0	7
Doctor appointed as responsible for contact with CDC and for referral.	0	0	0	7
Hospital doctors trained on TB disease and control	0	1	0	7
Health education activities organized by hospital doctors in campus*	1	2	0	7

* During the two study periods excluding the first month, i.e. Feb-July 2007 and Sep 2007-Jan 2008.

Note: N stands for the number of university hospitals. Two universities in the intervention group have two hospitals.

### Referral and tracing of TB suspects among university students

Before the intervention, there were no significant differences in referral rate, referral arrival rate, tracing rate, tracing arrival rate, and total arrival rate between the control group and the intervention group. After the intervention, all rates were significantly higher in the intervention group compared to the control group, except for the tracing arrival rate (69.2% vs. 45.8%, p = 0.31) (Table 4). When comparing the control group before and after the intervention, there were no differences in these five rates, while the intervention group showed significant increases in all rates except in the tracing arrival rate compared to before the intervention (37.0% vs. 69.2%, p = 0.06, data not shown). During the baseline period, 48.9% (43/88) and 45.7% (42/92) (p = 0.67) of university student TB suspects in the control and intervention group, respectively, arrived and were managed at the CDC. After the intervention, 46.2% (30/65) and 93.2% (68/73) (p < 0.001) of TB suspects were managed by the CDC, respectively (Table 4). Among all 176 university student TB suspects who arrived at the CDC during the study period, 16 (9.1%) (data not shown) were diagnosed with smear-positive TB.

**Table 4:** Referral, tracing and arrival rates in the control and intervention group before and after implementation of intervention measures

		Registered TB suspects	Referred TB suspects	Referral rate	Referral arrival	Referral arrival rate	Direct arrival within 3 days	TB suspects to be traced	TB suspects traced	Tracing rate	Tracing arrival	Tracing arrival rate	TB suspects arrived	Total arrival rate
		n	n	%	n	%	n	n	n	%	n	%	n	%
		A	B	C	D	E	F	G	H	I	J	K	L	M
Before	Control	88	36	40.9	16	44.4	16	56	26	46.4	11	42.3	43	48.9
	Intervention	92	41	44.6	17	41.5	15	60	27	45	10	37.0	42	45.7
	P-Value			0.62		0.79				0.88		0.69		0.67
After	Control	65	29	44.6	13	44.8	6	46	24	52.1	11	45.8	30	46.2
	Intervention	73	71	97.2	59	83.1	0	14	13	95	9	69.2	68	93.2
	P-Value			<0.001		<0.001				<0.001		0.31		<0.001

Note: C = B/A*100%, E = D/B*100%, G = A-D-F, I = H/G*100%, K = J/H*100%, L = D + F + J, M = L/A*100%

Non-arrival at the CDC in the control group and before the intervention in the intervention group was mainly due to TB suspects being referred to a general or TB hospital (72/130 = 55%) or sent home for treatment (26/130 = 20%) (Table 5). After the intervention, only 5 out of 73 TB suspects did not arrive at the CDC. Four of them were referred to a TB hospital and one went home.

**Table 5:** Reasons for non-arrival of TB patients at the CDC

				Reasons for patients not to arrive at the CDC						
		Number of patients registered	Number of patients not arrived	Treated at TB hospital	Left university for treatment in hometown	Treated at general hospital	Treated at another CDC within the district	Treated at university hospital	Denial of TB suspect status	Unknown
Before	Control	88	45 (51.1)	18	10	7	2	2	1	5
	Intervention	92	50 (54.3)	20	11	8	3	3	1	4
After	Control	65	3 (53.8)	11	5	8	2	4	2	3
	Intervention	73	5 (6.8)	4	1	0	0	0	0	0

## Discussion

The coverage of DOTS can be improved by implementing PPM cooperation, which enhances the collaboration between the different public and private health institutes involved in TB management in order to increase TB case detection and cure rates [10]. In some Asian countries, the proportion of TB patients who seek health care in private health institutes is high [11]; Therefore, some high TB burden countries such as India, Nepal, Vietnam, and the Philippines have developed public-private mix cooperation mechanisms, some of which resulted in great increases in case detection [12,13].

In China 70%-80% of TB patients seek help first in public general medical services while the proportion of patients who go to private clinic is low [14]. So activities to increase DOTS coverage in China should focus mainly on cooperation within the public sector, between CDC and public general medical services (public-public mix DOTS). The results of a review on 16 intervention studies in China focusing at general hospitals showed that referral of TB patients to the CDCs did improve as a result of interventions, and thereby did improve case detection and management of TB patients [15].

In China, there are many public medical services that are not managed by the health administration of the Ministry of Health, such as hospitals in prisons, armies, and universities. Patients in these places would seek health care first in related hospitals. These are public medical services that are rarely in contact with the CDC. TB suspects and patients are not referred systematically and usually are not treated according to NTP guidelines. We developed a cooperation mechanism between CDCs and university hospitals to increase case referral, arrival and tracing in order to improve the management, this includes diagnosis and treatment of TB under DOTS programs.

Through the study intervention measures, the university hospital doctors learned (more) about the NTP and DOTS strategy and the importance of referral of patients to the CDC for standardized management, and they could communicate this knowledge to TB suspects whom they referred. This has led to great improvements in referral and arrival of TB suspects among university students in the intervention group. The referral rate, the referral arrival rate, tracing rate and total arrival rate increased to 97.2%, 83.1%, 95.0%, and 93.2% respectively, while the rates remained between 40% and 50% in the control group. After the intervention, 93% of suspects arrived at the CDC for TB diagnosis and treatment versus less than 50% before the intervention and in the control group. Nine percent of the TB suspects were diagnosed with smear-positive TB. Studies in rural areas in China, with more poverty and less easy access to medical TB service, observed higher proportions of patients with smear-positive TB (unpublished data). In this study among more affluent and educated university students with easy access to health care, symptomatic patients will seek health care at an earlier stage.

Only the tracing arrival rate did not improve satisfactory, although it did clearly improve, from 37.0% before to 69.2% after the intervention was introduced. According to university regulations TB cases should be hospitalized or go back home for treatment (managed by the local TB dispensary) in order to prevent spread of TB in the university. Our results confirm that this seems to be the reason why some of the referred TB suspects did not arrive at the CDC within the university district, especially during the baseline period.

We achieved bigger improvements than observed in other studies on the effect of PPM activities in non-student populations [16-21]. This may be the result of the practical, operational design of our intervention. This probably led to higher compliance with the guidelines and procedures introduced in the university hospitals than in the institutions involved in the other studies. Another research study also showed the usefulness of adapting generic TB control guideline into an operational guideline for county level TB doctors [22]. Also it may have helped that patients in this study have a high level education, usually have a student insurance covering medical expenses and that are good transportation possibilities to reach the CDCs in these university cities are available.

Besides these strengths, there were also some limitations of our study. Because of limited funding, the number of included TB suspects per group was quite low and our evaluation was conducted five months after the intervention was introduced. As a result, the full effects may not have been reached yet. Also, we were not able to assess the long term effects.

Improved referral was crucial in this PPM cooperation mechanism [23]. Because the arrival of TB suspects registered by the university hospital at the CDC within three days after registration increased, the proportion of registered TB suspects that needed to be traced decreased from 54.3% (= 50/92) to 6.8% (= 5/73) (Table 4). This relieved human, material and financial resources in generally overburdened CDCs. This in turn may have led to the increased tracing rate of suspects. Also, improved referral will diminish diagnostic delay, which may lead to improved treatment outcomes.

## Conclusion

We showed that it is feasible to set up a PPM cooperation mechanism between CDC and university hospitals in order to increase the total arrival rate of TB suspects at the CDC where they will be managed under DOTS conditions. The main focus was on intervening at referral by university hospitals. However, we showed that this intervention had effects also on tracing and arrival rates of TB suspects. As a next step, we recommend that the PPM cooperation should be widened to include all university hospitals in China.

## Abbreviations

TB: Tuberculosis; DOTS: directly observed treatment-short course; PPM: public-public mix, tuberculosis control strategy recommended by the World Health Organization; NTB: National TB Program; CDC: Centers for Disease Control and Prevention

## Competing interests

The authors declare that they have no competing interests.

## Authors' contributions

TZ, LG and SZ designed the protocol, carried out the field work, did data analysis and drafted the manuscript. WL and GC participated in the write up of the protocol. WL, GC, MH carried out the field work, performed the data collection, participated in the data analysis and write up of the manuscript. GH, MW and SH participated in the write up of the protocol, in the data analysis and write up of the manuscript. MW and SH revised and commented on the draft. All authors read and approved the final manuscript.

## Author's information

TZ

Deputy Director of Shaanxi Provincial Institute for TB Control and Prevention

Deputy Dean of Shaanxi Provincial academe for TB Control and Prevention

Secretary-General of Shaanxi province association against Tuberculosis

Associate senior doctor

He has undertaken several tasks related to TB control respectively in clinical TB treatment, health education, and research in the last 24 years.

## Pre-publication history

The pre-publication history for this paper can be accessed here:

http://www.biomedcentral.com/1471-2458/11/147/prepub
